# Sponge Communities on Caribbean Coral Reefs Are Structured by Factors That Are Top-Down, Not Bottom-Up

**DOI:** 10.1371/journal.pone.0062573

**Published:** 2013-05-08

**Authors:** Joseph R. Pawlik, Tse-Lynn Loh, Steven E. McMurray, Christopher M. Finelli

**Affiliations:** Department of Biology and Marine Biology, Center for Marine Science, University of North Carolina Wilmington, Wilmington, North Carolina, United States of America; Victoria University Wellington, New Zealand

## Abstract

Caribbean coral reefs have been transformed in the past few decades with the demise of reef-building corals, and sponges are now the dominant habitat-forming organisms on most reefs. Competing hypotheses propose that sponge communities are controlled primarily by predatory fishes (top-down) or by the availability of picoplankton to suspension-feeding sponges (bottom-up). We tested these hypotheses on Conch Reef, off Key Largo, Florida, by placing sponges inside and outside predator-excluding cages at sites with less and more planktonic food availability (15 m vs. 30 m depth). There was no evidence of a bottom-up effect on the growth of any of 5 sponge species, and 2 of 5 species grew more when caged at the shallow site with lower food abundance. There was, however, a strong effect of predation by fishes on sponge species that lacked chemical defenses. Sponges with chemical defenses grew slower than undefended species, demonstrating a resource trade-off between growth and the production of secondary metabolites. Surveys of the benthic community on Conch Reef similarly did not support a bottom-up effect, with higher sponge cover at the shallower depth. We conclude that the structure of sponge communities on Caribbean coral reefs is primarily top-down, and predict that removal of sponge predators by overfishing will shift communities toward faster-growing, undefended species that better compete for space with threatened reef-building corals.

## Introduction

Food chain dynamics is considered a central theory in ecology [1], and proposes that populations of organisms that make up communities are controlled by processes that are bottom-up (nutrients, food) or top-down (predation). The relative effect of these two processes has been the subject of considerable debate [2], but in most cases, a greater understanding of the complexity of an ecosystem reveals that both are important [3], [4], [5], [6], [7]. Ecosystem functioning has gained renewed interest [8], [9] as regulatory agencies increasingly adopt ecosystem-based management strategies, particularly for marine systems [10], [11], [12].

Coral reefs of the Caribbean region have undergone a marked transformation as reef-building corals have declined due to multiple stressors including disease, temperature extremes, storm damage, and the loss of key herbivores [13]. Macroalgae now cover the greatest surface area on many reefs [14], and while arborescent gorgonian corals are often visually dominant [15], sponges have become a primary component of Caribbean coral reef ecosystems [16]. In addition to their large biomass on shallow-water reefs, sponges dominate light-limited reef interstices, caves and mesophotic reefs [17], but are also found in grassbed, hardbottom, and mangrove habitats [18]. Moreover, recent evidence indicates that sponge populations on Caribbean reefs are increasing [16].

Marine sponges are primarily suspension-feeding organisms that derive their food from picoplankton, the size category that includes bacteria and prochlorophytes. Two recent studies concluded that the availability of picoplanktonic food was of principal importance in structuring sponge communities on Caribbean coral reefs [19], [20], concluding that “most of the variability in their distribution and abundance, from reef to reef, and with depth, can be explained principally by bottom-up processes” ([19], p. 286). The first study compared 3 tube sponge species at different reef sites and found that tubes were longer and elongated faster at greater depth, which correlated with higher abundances of food, mostly prochlorophytes and heterotrophic bacteria, on deeper reefs [19]. In a companion study, the tube sponge C*allyspongia vaginalis* was transplanted to shallow and deep sites (12 m and 25 m) on Conch Reef, off Key Largo, Florida, and exhibited greater growth at greater depth, again correlated with higher picoplankton abundances on deeper reefs [20]. These data were corroborated by *in situ* measurements of sponge respirometry and pumping rates at deep and shallow sites, which were combined with flow cytometry measurements of food availability to construct energetic budgets for sponges at each depth that indicated a greater scope for growth for sponges at the deep site where food availability was higher [20].

Research on the chemical ecology of Caribbean reef sponges supports an alternative hypothesis that predatory fishes (mostly angelfishes and parrotfishes) play an important role in controlling the structure of sponge communities through differential predation on chemically undefended sponges [21], [22], [23]. Many sponge species on reefs produce distasteful secondary metabolites in their tissues that deter feeding by fish predators [24], [25], [26], [27], [28], while other species persist despite their lack of a chemical defense. When pieces of branching sponges in these two categories were attached to the reef inside and outside of predator-excluding cages, defended sponges grew slower and were unaffected by predation, while undefended sponges grew faster inside, but were grazed by predators outside, resulting in a slower accumulation of biomass [29]. Not only have these caging experiments provided evidence of a resource trade-off between the production of chemical defenses and rapid growth, they also suggest that sponge communities are composed of slow-growing species that are chemically defended from predation and faster-growing species that are not, with the relative abundance of each type on the reef determined by predation pressure (i.e. top-down control).

We tested the relative importance of bottom-up and top-down processes on sponge growth by performing predator-exclusion experiments at both shallow (15 m) and deep (30 m) sites on Conch Reef, off Key Largo, Florida. The site of the undersea research station *Aquarius* for two decades [30], Conch Reef is one of the best studied coral reefs in the world [31]. Deep-water internal waves break over Conch Reef, bringing picoplankton-rich water to deeper portions of the reef for greater periods of time than shallower portions [32]. The physical oceanography of this reef is very well described [33], and it was the location of previous studies that concluded that bottom-up effects played a predominant role in sponge growth [19], [20], but these studies did not consider the impact of predation. We combined our test of the relative importance of bottom-up and top-down processes with a comparison of the growth of both chemically defended and undefended sponge species to further assess the importance of resource trade-offs between chemical defenses and growth among Caribbean sponges.

## Materials and Methods

### Caging Experiments

All experiments were conducted along a depth gradient running west to east ∼100 m south of the *Aquarius* undersea research laboratory on Conch Reef, off Key Largo, Florida (24°57′00″N, 80°27′13″W). This is the same site and depth profile as previous surveys and experiments that concluded that food abundance (picoplankton concentration) was the primary factor structuring sponge communities on Caribbean reefs [19], [20].

The first experiment was conducted with 4 branching sponge species for 287 days (18 Aug 2010–1 Jun 2011). Branching sponges were chosen for the first set of experiments because sponges of this morphology are adapted to breakage and reattachment (fragmentation) as a form of asexual reproduction when exposed to high water flow from storm events or currents, so they easily survive and grow after manipulation, including brief removal from water for determining wet mass in the lab [29], [34]. We collected, tagged and weighed pieces of two chemically undefended species (*Callyspongia armigera, Iotrochota birotulata*) and two chemically defended species (*Amphimedon compressa, Aplysina cauliformis*) [24]. The tissues of *Amphimedon compressa* contain predator-deterring pyridinium alkaloids, primarily amphitoxin [35], while *Aplysina cauliformis* produces distasteful brominated tyrosine derivatives, primarily fistularin-3 [36]. A second experiment using the same methods was performed with *Callyspongia vaginalis*, the same species of tube sponge used in previous studies demonstrating bottom-up effects [19], [20] for a period of 328 days (18 June 2011–11 May 2012).

Experimental methods were the same as previously used over 9 years to compare growth rates of chemically defended and undefended sponges of different species at shallower depths on nearby reefs [29]. Sponge pieces used in experiments were collected from 10–30 m depth at nearby reefs (Conch Wall, N. Dry Rocks Reef) that were outside research-only areas where collections were prohibited. Sponge pieces were cut with a scalpel to a length of ∼10 cm (∼50 g wet mass), brought into the laboratory in clean, aerated seawater, wet mass determined on an electronic scale, a unique tag on a zip-tie was attached to each piece, and all pieces returned to the field within 4 h. Sponge pieces were distributed haphazardly on plastic plates that were affixed to 0.3×0.6 m plastic mesh (2.5 cm^2^ holes) bases that were anchored to the limestone reef with galvanized nails topped with 5 cm washers. On each mesh base, two sponge pieces on plastic plates were attached with zip-ties, one ∼15 cm from each short edge of the rectangular base, along the center axis. One of these two sponges was haphazardly chosen to be covered with a cube-shaped mesh cage top, 0.3 m on each side. At each depth location (15 m and 30 m), 20 sponge pieces of each species were placed both inside and outside of cages. Biofouling on cages was minimal during the experimental period, and was similar across sites. At the end of the experiment, sponges were carefully removed from plastic plates and mesh bottoms with their individual tags intact and brought back to the laboratory (often in individual zipped bags), where they were re-weighed. Many of the labor-intensive portions of this experiment, particularly deploying the mesh bases and cages, were performed using saturation diving from the undersea habitat *Aquarius*.

There has been considerable debate in the marine ecology literature about technical issues involved in caging experiments [37]. As previously discussed [29], we did not include cage controls in these experiments (a third treatment in which cages have one or more sides left open) because the results of past experiments showed they were unnecessary (no difference in the growth of defended species inside and outside of cages). Moreover, while cages may have some effect of altering flow around suspension-feeding sponges that could reduce feeding capability, this effect would be in the opposite direction from the outcome expected for undefended sponge species (caged sponges will not be grazed and should grow more); therefore, enhanced growth of caged versus uncaged sponges would be a conservative result.

### Hydrographic Data

Temperature, current speed and current direction data were collected near continuously at 20 m depth on Conch Reef for 22 Aug 2010–1 Jun 2011 using an InterOcean S4 current meter. Additionally, temperature data were collected at 15 m and 30 m for 18 June 2011–11 May 2012 using Onset HOBO Water Temp Pro loggers. Data were collected every minute over five-minute intervals every 10 minutes.

### Surveys of Benthos and Fishes

Surveys of the benthic community and spongivorous fish abundance were carried out at 15 m and 30 m on Conch Reef, Florida. At each depth, five 20-m line transects were laid in a single file, with each 20 m line separated by a 5 m gap. The reef bottom was sampled using five evenly spaced 1×1 m^2^ quadrats per transect line, with the benthos under 25 points within each quadrat recorded, for a total of 625 points per depth. The benthic categories recorded were sponge, hard corals, gorgonians and macroalgae. Spongivorous fishes were counted along the same 5 transect lines as the benthic surveys using the Reef Check survey method (see [38] for description of method and list of known spongivorous fish species).

### Statistical Analyses

Sponge growth data were transformed prior to statistical analyses, as in a previous study [29], but without the time component because the duration of the experiment for the 4 branching sponge species was the same. Growth index (final wet mass/initial wet mass) was compared between caging treatments and depth using a 2-way ANOVA for each branching sponge species in the program JMP 7.0 (SAS Institute). With the exception of *Amphimedon compressa*, growth indices for the other 3 branching species were log transformed to reduce heteroscedasticity in sample variance. For the tube sponge *Callyspongia vaginalis*, the growth index was compared between depths for the uncaged sponges only (cages were lost at the 15 m site following winter storms), and the caging effect was only analyzed for the sponges at 30 m. Growth indices were log transformed and compared in separate Student’s t-tests for depth and caging. The proportional cover of hard coral, sponges, and macroalgae was calculated for each 20 m transect, arc-sine transformed and compared between 15 and 30 m using the t-test. Abundances of angelfishes and parrotfishes within 500 m^3^, the volume surveyed along each 20 m transect, were also compared between 15 m and 30 m using the t-test.

Sponge collections, caging experiments, and surveys of fishes and benthos were carried out under a permit from the Florida Keys National Marine Sanctuary (FKNMS-2009-126-A1), with the caging experiments and surveys conducted within the Conch Reef Special Protected Area.

## Results

**Figure 1:**
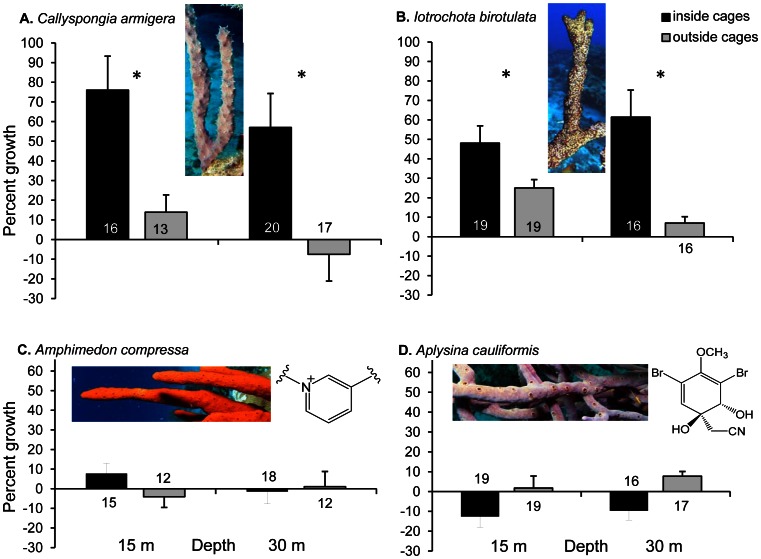
Percentage growth of branching sponge pieces (change in wet mass) 287 days after attachment inside and outside of predator-excluding cages at 15 and 30 m depth on Conch Reef, Florida. A: *Callyspongia armigera* and B: *Iotrochota birotulata* lack chemical defenses, while C: *Amphimedon compressa* and D: *Aplysina cauliformis* contain alkaloids that deter fish predators, represented by a portion of the chemical structure of amphitoxin for the former and aeroplysinin-1 for the latter. Surviving number of 20 replicates is shown for each bar, error bars are standard error. Statistical analyses were performed on transformed data (growth index). An asterisk indicates a significant difference in growth inside vs. outside cages (*p*<0.01).

Regardless of depth, the two chemically undefended branching sponge species grew significantly more inside cages that excluded predatory fishes, with no significant interactions between depth and caging (Fig. 1, *Callyspongia armigera*: F = 19.94, df = 1, *p*<0.0001, *Iotrochota birotulata*: F = 10.80, df = 1, *p* = 0.0016). Bite marks were observed on many of the pieces of chemically undefended species outside of cages, despite rapid rates of healing for these species [39]. Greater growth was observed for *Callyspongia armigera* at 15 m vs. 30 m (F = 4.97, df = 1, *p* = 0.0294), and growth was similar between depths for the other 3 branching sponge species. Chemically defended sponge species grew relatively little compared to undefended species (<10% vs. ∼45–75% inside cages). Growth was not significantly different whether defended sponges were caged or exposed to predators (Fig. 1).

**Figure 2:**
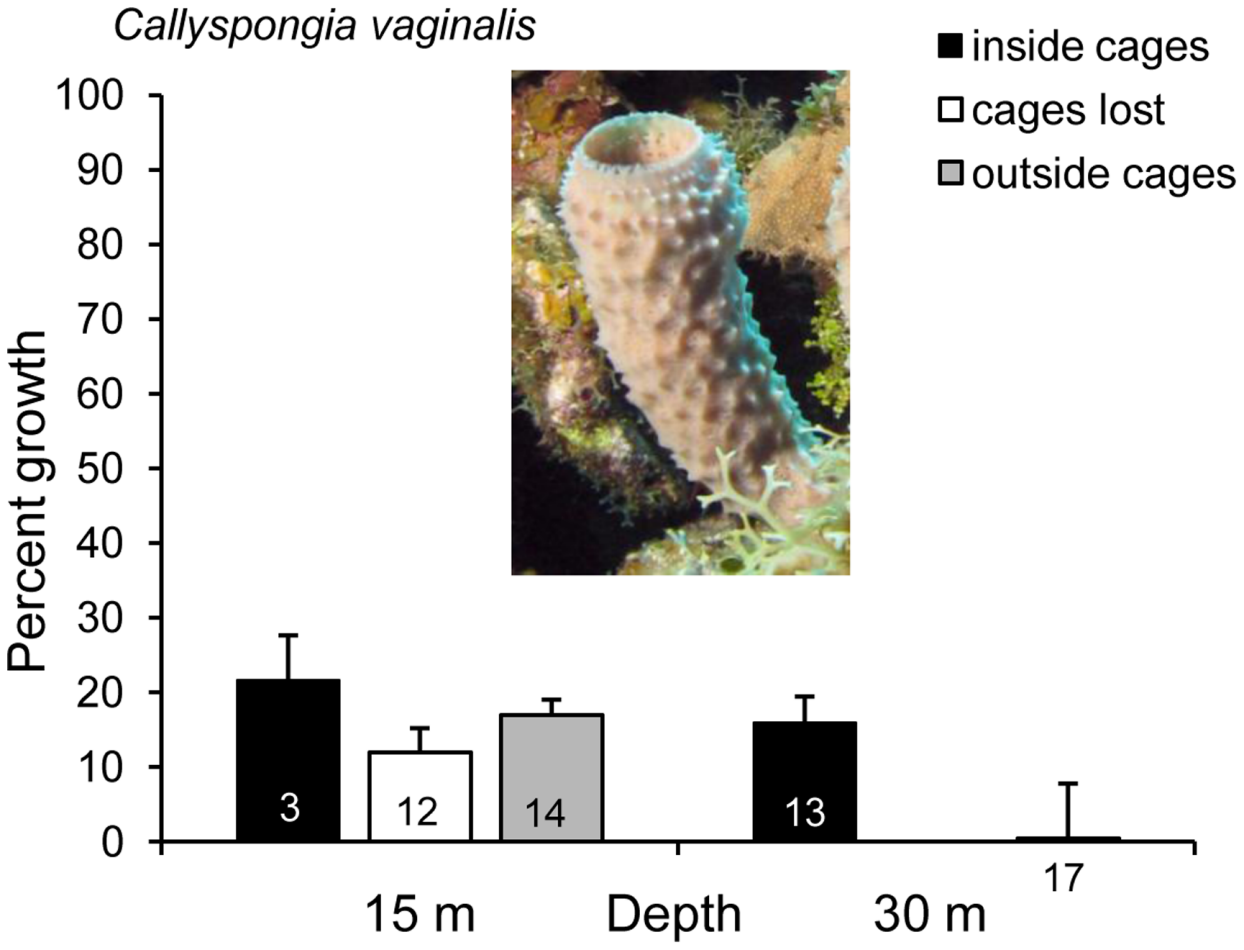
Percentage growth of the gray tube sponge *Callyspongia vaginalis* (change in wet mass) 358 days after placement inside and outside of predator-excluding cages at 15 m and 30 m depth on Conch Reef, Florida. Winter storms removed all but 3 cages at the 15 m site; growth of sponges that had been caged by lost cages is shown separately. Surviving number of 20 replicates is shown for each bar, error bars are standard error. Statistical analyses were performed on transformed data (growth index).

During the second iteration of the experiment using the tube sponge *Callyspongia vaginalis*, winter storms removed all but a few cages at the shallow site. Despite the loss of cages, growth of the remaining sponge tubes was greater at the shallow than at the deep site for uncaged sponge tubes (Fig. 2, 1-tailed Student’s t-test, t = −2.20, df = 29, *p* = 0.0180), and the effect of caging at the deep site was significant, with virtually no sponge growth outside of cages (Fig. 2, t = −1.72, df = 28, *p* = 0.0486). Again, bite marks were observed on many of the pieces of *C. vaginalis* outside of cages.

Data from temperature sensors on Conch Reef showed similar hydrography to previous years, including a data set from 2000–2005 that was recently published [40]. These measurements confirmed the periodic arrival of cold-water nutrient transport events that increase in magnitude and duration with increasing depth, providing higher concentrations of picoplankton to deeper sites, all of which is well described for Conch Reef [20], [32], [33], [40]. Additionally, analysis of water samples taken at both depths in December 2011 using flow cytometry confirmed that there was an ∼1.5 fold enhancement of picoplankton (see [20], Fig. 2) at 30 m compared to 15 m (McMurray, unpublished data), validating previous studies that have described this persistent hydrographic feature at Conch Reef using chlorophyll signatures [32], [33].

**Figure 3:**
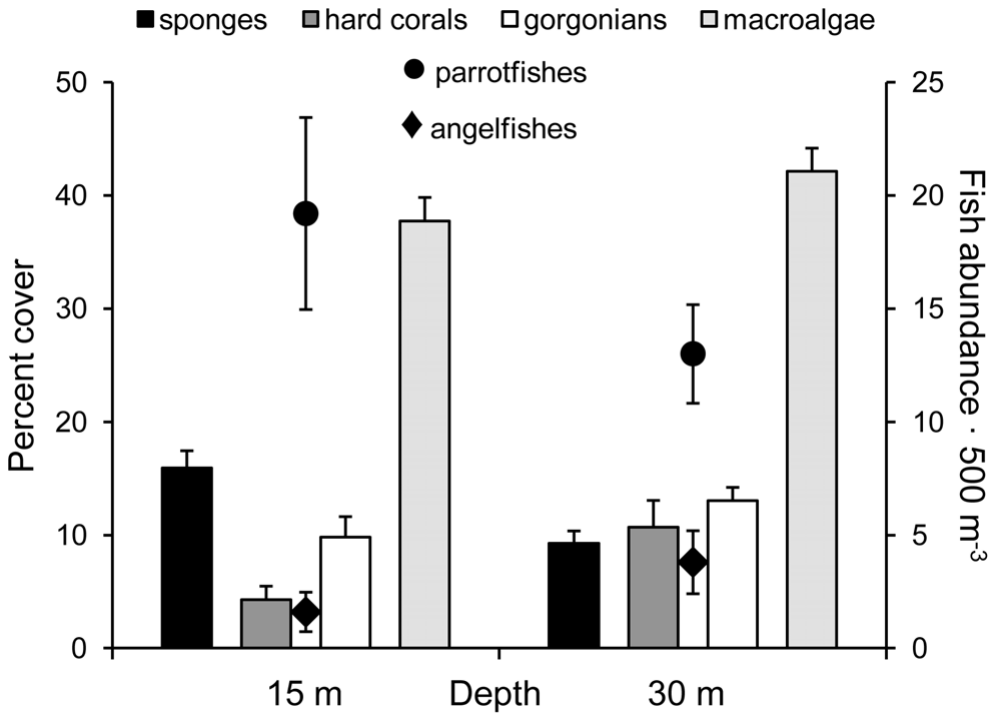
Percentage benthic cover of sponges, reef-building corals, gorgonian corals and macroalgae and abundance of the dominant sponge-eating fishes at 15 m and 30 m depth on Conch Reef, Florida. Benthic cover is shown in bars (y-axis on left), fish abundance in dots and diamonds (y-axis on right). Error bars are standard error.

Surveys of benthic organisms and of sponge-eating fishes at depths of 15 m and 30 m on Conch Reef revealed overall higher sponge cover at the shallower depth (Fig. 3). Abundances of angelfishes and parrotfishes were not significantly different at shallow and deep sites (Fig. 3, t-test, *p*>0.05). Sponge cover was greater at the shallower site (16.0% vs 9.3%; *t*-test, *t* = −3.61, df = 8, *p* = 0.0034), with about the same cover of chemically defended sponges at each site (62.5% vs. 57.6%) and the remaining cover at each site split between chemically undefended and variably defended species. Cover of reef-building corals was greater at the deeper depth (Fig. 3, *t*-test, *t* = 2.41, df = 8, *p* = 0.0212), while gorgonian and macroalgal cover was not significantly different between 15 m and 30 m (Fig. 3).

## Discussion

The results of our experiments do not support bottom-up control of sponge communities on Conch Reef, a site where higher concentrations of picoplanktonic food at deeper sites is well established, because sponges in predator-excluding cages at the deeper site either exhibited no difference in growth (3 species) or grew less (2 species) than caged sponges at the shallower site. The absence of a bottom-up effect on sponge growth at Conch Reef was surprising, given that most ecosystems that have been studied show evidence of both top-down and bottom-up processes in structuring communities [5], [7], and because the two previous studies of sponges at the same research location concluded that bottom-up effects were dominant [19], [20]. The first of these looked at differences in the length and elongation of tube sponges as a function of depth, but these methods are problematic because sponges grow in many dimensions besides elongation, including wall thickening and the addition of new tubes [41]. Possible explanations for longer tube sponges at greater depth include less of an impact of storm-related currents at depth, which tend to rip sponges off the substratum in shallower water and result in older, longer tubes at greater depth, and differences in tube thickness and colony formation (number of tubes) because of flow differences in shallow and deep water [42]. Our own observations confirm a conspicuous removal of non-recumbent sponges by storm surge during the second iteration of the experiment that resulted in the loss of cages at the shallow site (Fig. 2), with many branching and tube-shaped sponges unattached and dying in the sand channels between reefs.

The second study that concluded that bottom-up effects primarily influenced sponge growth [20] did so on the basis of growth experiments with a single species of tube sponge, *Callyspongia vaginalis*, which is a primary food item of sponge-eating fishes [21], [43]. In the first iteration of the experiment designed for the present study, we used the closely related branching sponge, *Callyspongia armigera*, which, like *C. vaginalis*, is chemically undefended [24], and observed significantly greater growth of this species at the shallower depth (Fig. 1A). We repeated the experiment using *C. vaginalis* a year later, but winter storms removed all but a few cages at the shallow site. Nevertheless, growth of the remaining sponge tubes was greater at shallow than at deep sites for uncaged sponge tubes (Fig. 2), and the effect of caging at the deep site was significant, with virtually no sponge growth outside of cages due to predation. Taken together, these results are also contrary to bottom-up control of sponge growth and support the hypothesis that top-down effects structure the sponge community. The enhanced growth of *C. vaginalis* at the deep site previously observed by Trussell et al. [20] may have been due to lower levels of predation at that site over the period of their experiment, considering that no exclosures were used to prevent sponge predation. In previous caging experiments, we demonstrated that the tube sponge *C. vaginalis* grows more slowly than the branching *C. armigera*, but that the former produces many more larvae than the latter, evidence of an additional resource trade-off between growth and larval production [34].

Other recent studies, also at Conch Reef, have not supported bottom-up control of sponge growth. There was no significant difference in the growth of the dominant habitat-forming organism, the giant barrel sponge *Xestospongia muta*, at 15 m, 20 m, and 30 m depths using digital image analysis of 104 tagged sponges over 4.5 years [41]. Further, density of *X. muta* was greater in plots at 15 m and 20 m depth than at 30 m [16]. Also contrary to the bottom-up hypothesis, data from our surveys of benthic organisms and of sponge-eating fishes at 15 m and 30 m revealed overall higher sponge cover at the shallower depth (Fig. 3). Abundances of angelfishes and parrotfishes were not significantly different at shallow and deep sites (Fig. 3), indicating that despite similar levels of predation on a mixed population of chemically defended and undefended sponges, sponge cover was greater at the shallower site, where food availability was lower than at the deep site. This modest difference is probably due to a disproportionate effect of sponge consumption by large angelfishes, which were more abundant at the 30 m site (Fig. 3) and, unlike parrotfishes, primarily eat sponge tissue [43], [44]. Alternatively (or in addition), higher sponge cover at 15 m could be due to greater light levels at shallower depths, as some sponge species have photosynthetic microbial symbionts that enhance sponge growth [45]. The only phototrophic sponge used in the present study was *Aplysina cauliformis*, which exhibited negative growth inside cages at both 15 m and 30 m, although this effect was not significant when compared to the minimal growth of sponges outside of cages for this slow-growing, chemically defended species (Fig. 1D). Interestingly, this species grew much faster in similar experiments conducted at 7 m [29], suggesting that the high light levels found on very shallow reefs may promote the growth of some phototrophic species [46].

Overall, experimental and distributional data suggest that the growth of sponges is not limited by food availability based on experiments and surveys at Conch Reef, where the parameters regarding picoplankton distribution and supply as a function of depth is well characterized. But are these data generally applicable for coral reefs across the Caribbean? For several reasons, we believe they are. First, Conch Reef is unusual in its topography and hydrodynamics in having the “plankton pump” of internal waves bringing greater levels of picophytoplankton from deeper water up the steep reef slope to oligotrophic shallow water [32]. Most Caribbean reefs have a more gradual slope and lack the same level of hydrodynamic forcing; hence, if a bottom-up effect on sponge growth is likely to be demonstrated for any coral reef in the Caribbean, it should be evident at Conch Reef, but it is not. In fact, identical sponge growth experiments conducted on a shallow reef (7 m) more typical of the Caribbean with a very gradual reef slope yielded similar or higher rates of sponge growth for all 4 of the branching species used in the present study [29]. Second, sponge community composition is remarkably homogeneous on reefs across the Caribbean [22]; specifically, sponge diversity and abundance at Conch Reef is similar to many reefs across the Caribbean (Loh and Pawlik, unpublished data forthcoming). Third, the diversity of sponge-eating fishes is also similar on reefs across the Caribbean, although the abundance of these predators varies as a function of human fishing activities, resulting in a range from highly protected (Bonaire, Cayman Brac, Exuma Cays) to heavily fished (Jamaica, Martinique), with Conch Reef falling between them (Loh and Pawlik, unpublished data forthcoming). Therefore, the combination of region-wide similarities in sponge ecology with the lack of evidence for food limitation from manipulative experiments at sites that span the hydrographic diversity of reefs in the region provide a compelling argument against bottom-up effects having an important role in structuring sponge communities on Caribbean coral reefs.

Although the results of the present study support the conclusion that top-down effects primarily structure sponge communities on Caribbean coral reefs, there are certainly other factors, particularly abiotic ones, that affect recruitment, growth, and sponge community development. Just as temperature extremes are known to limit sponge distributions in mangrove habitats [18], [47], temperature fluctuations can completely alter reef sponge communities [48]. High water flow events, such as those generated by storm surge, may depopulate non-recumbent sponges (as observed during the second iteration of the experiments reported here) or may generally prevent some species of sponges from recruiting, as on the windward side of many islands and atolls. More broadly, ocean currents could restrict the dispersal of sponge recruits, although this is not an important factor for the well-mixed Caribbean region [49]. Sponges require water flow to suspension-feed, and may grow faster when exposed to higher flow. One recent study demonstrated that pieces of *Amphimedon compressa* and *Iotrochota birotulata* (two of the same species used herein) grew faster when suspended higher in the water column above the reef, as when these branching species grow upward or attach onto other sponges or gorgonian corals [50]. Interestingly, this study reported faster growth for *A. compressa* than *I. birotulata,* but did not incorporate a caging component to remove the effect of predation on either species.

Although sponge cover was greater at the shallower site on Conch Reef, the reverse was true for the cover of reef-building corals (Fig. 3). This pattern is opposite that expected for hard corals, which rely on photosynthetic symbionts for growth, and may reflect higher rates of corallivory by parrotfishes or more frequent heat stress events at shallower depths, but may also represent enhanced competition between sponges and corals at shallower sites. Macroalgal cover might also be expected to decrease with depth, but there was no difference between sites in our surveys (Fig. 3).

The sponge community on Caribbean coral reefs provides a remarkably simple system for testing ecological theory on foodweb dynamics and resource allocation, particularly when compared to more commonly studied terrestrial plant-herbivore communities that are complicated by variations in abiotic factors such as light, rainfall, nutrients, and soil chemistry, as well as the heterogeneity resulting from limited dispersal and allopatric speciation [22]. The present study demonstrates that, rather than a complex or context-dependent combination of bottom-up and top-down effects, predation is the primary determinant of sponge community structure on Caribbean coral reefs. As a clear example of alternative resource allocation, chemically defended sponges are largely unaffected by predation, but heal wounds [39], grow [29], and recruit [51] at slower rates than chemically undefended sponge species, which are not protected by structural defenses [52], [53], and are subject to fish grazing. While branching sponge species are preferred subjects for manipulative experiments, recumbent and encrusting species often dominate substratum coverage, and this morphological group is also made up of both chemically defended and undefended species [21], [24]. Sponges are often competitively dominant over reef-building corals, and one of the most common Caribbean sponges, *Mycale laevis*, a chemically undefended, recumbent species, will smother adjacent corals on reefs where sponge-eating fishes have been removed by overfishing [38], [54]. We predict that overfished reefs that lack spongivores will become dominated by faster-growing undefended sponge species, which better compete for space with reef-building corals. This has important implications for fisheries management across the Caribbean, as some coral species are already listed as “critically endangered” on the IUCN Red List, with 4 reef-building Caribbean species on the top ten list for extinction risk [55]. Sponge populations are already increasing on Caribbean reefs [16], [56], and as the impacts of climate change and ocean acidification further disrupt marine communities [57], it seems likely that reef-building corals and some macroalgae will suffer greater harm than sponges, which do not form limestone skeletons [58]; hence, Caribbean reefs of the future are likely to become increasingly dominated by sponges.
